# Draft Genome Sequence of the Aquatic Fungus *Hymenoscyphus tetracladius* Strain NNIBRFG329

**DOI:** 10.1128/MRA.01168-18

**Published:** 2018-11-29

**Authors:** Jaeduk Goh, Hye Yeon Mun, Yoosun Oh, Namil Chung

**Affiliations:** a Fungal Resources Research Division, Nakdonggang National Institute of Biological Resources, Sangju, Republic of Korea; Vanderbilt University

## Abstract

Hymenoscyphus tetracladius (anamorph, Articulospora tetracladia) is a common aquatic hyphomycetous fungus. Here, we report the draft genome sequence of H. tetracladius strain NNIBRFG329, which comprises 41.8 Mb in 20 scaffolds with an overall G+C content of 46.95% and an *N*_50_ value of 3.973 Mb.

## ANNOUNCEMENT

Hymenoscyphus tetracladius (anamorph, Articulospora tetracladia) is well known as one of the aquatic fungi that contribute to leaf decomposition in streams (1, 2). This fungus belongs to the family Helotiaceae (order Helotiales, phylum Ascomycota) and produces an unusual form of asexual spores suitable to attach on the leaf surface. Leaf decomposition by aquatic fungi such as H. tetracladius is important for material recycling and the food chain in freshwater ecosystems (3). In South Korea, strain NNIBRFG329 was first reported as Articulospora tetracladia (4). Many studies of this fungus have focused on ecology, classification, and evolution, but there have been few genetic or genomic studies of this fungus. Here, we report the first draft genome sequence of Hymenoscyphus tetracladius NNIBRFG329, one of the common aquatic fungi.

NNIBRFG329 was isolated from plant litter as previously described (5). Genomic DNA and total RNA were extracted from mycelia grown in potato dextrose broth by shaking at 150 rpm for 1 week at 20°C, using the DNeasy minikit (Qiagen, Valencia, CA) and the Easy-Spin total RNA extraction kit (iNtRON, Sungnam, Republic of Korea). The genome sequence of NNIBRFG329 was obtained through a combination of 4 PacBio RS II single-molecule real-time (SMRT) cells (total of 467,715 reads and 4,059,549,032 bp) and 1 paired-end (total of 49,840,740 reads and 5,033,914,740 bp) and 2 mate pair libraries (total of 87,568,452 reads and 8.84 Gb) on the Illumina HiSeq 2000 platform (Theragen Etex Bio, Suwon, Republic of Korea). The quality of the reads was checked using FastQC v0.10.1 (http://www.bioinformatics.babraham.ac.uk/projects/fastqc) and was preprocessed using Trimmomatic v0.30 for paired-end sequencing (6) and NextClip v1.3 for mate pair sequencing (7). The reads were assembled using Canu v1.6 for PacBio reads (8) and SOAPdenovo v2.04 for Illumina reads (9) and were merged using HaploMerger2 v.20151124 (10). The draft genome of H. tetracladius NNIBRFG329 consisted of 20 contigs with an *N*_50_ value of 3.973 Mb and 428.53-fold coverage. The total length of the assembled genome was 41,846,809 bp with a G+C content of 46.95%. The maximum and minimum scaffold lengths were 7,110,143 bp and 2,165 bp, respectively. This draft genome sequence will support the identification of genes related to leaf litter decomposition by aquatic fungi in freshwater ecosystems and contribute to genetic research studies of the fungi.

### Data availability.

The draft genome sequence of H. tetracladius NNIBRFG329 has been deposited in GenBank under accession number PYIV00000000. The SRA accession number is SRR7800875. This paper describes the first version of the genome.
